# Cost-effectiveness analysis of pemetrexed versus docetaxel in the second-line treatment of non-small cell lung cancer in Spain: results for the non-squamous histology population

**DOI:** 10.1186/1471-2407-10-26

**Published:** 2010-01-29

**Authors:** Yumi Asukai, Amparo Valladares, Carlos Camps, Eifiona Wood, Kaisa Taipale, Jorge Arellano, Alejo Cassinello, José Antonio Sacristán, Tatiana Dilla

**Affiliations:** 1 IMS Health, 7 Harewood Avenue, NW1 6JB, London, UK; 2 Eli Lilly SA, Avenida de la Industria 30, 28108 Alcobendas, Madrid, Spain; 3 Hospital General Universitario, Avenida Tres Cruces 2, 46014 Valencia, Spain; 4 Eli Lilly, Erl Wood Manor, Windlesham, Surrey GU20 6PH, UK

## Abstract

**Background:**

The objective of this study was to conduct a cost-effectiveness evaluation of pemetrexed compared to docetaxel in the treatment of advanced or metastatic non-small cell lung cancer (NSCLC) for patients with predominantly non-squamous histology in the Spanish healthcare setting.

**Methods:**

A Markov model was designed consisting of stable, responsive, progressive disease and death states. Patients could also experience adverse events as long as they received chemotherapy. Clinical inputs were based on an analysis of a phase III clinical trial that identified a statistically significant improvement in overall survival for non-squamous patients treated with pemetrexed compared with docetaxel. Costs were collected from the Spanish healthcare perspective.

**Results:**

Outcomes of the model included total costs, total quality-adjusted life years (QALYs), total life years gained (LYG) and total progression-free survival (PFS). Mean survival was 1.03 years for the pemetrexed arm and 0.89 years in the docetaxel arm; QALYs were 0.52 compared to 0.42. Per-patient lifetime costs were € 34677 and € 32343, respectively. Incremental cost-effectiveness ratios were € 23967 per QALY gained and € 17225 per LYG.

**Conclusions:**

Pemetrexed as a second-line treatment option for patients with a predominantly non-squamous histology in NSCLC is a cost-effective alternative to docetaxel according to the € 30000/QALY threshold commonly accepted in Spain.

## Background

Lung cancer is the leading cause of cancer deaths worldwide [1]. It is the most common tumour in Spain, found mainly in men, and has the highest mortality. In 2001, there were over 16000 cases of lung cancer diagnosed in men and 1900 cases in women [2].

In 2005, 16647 men and 2471 women died from lung cancer, accounting for 27% and 7%, respectively, of all cancer deaths in Spain [3]. In Spain median survival rates of 40 weeks have been reported for patients with non-small cell lung cancer (NSCLC) [4] and 5-year survival is poor, estimated at only around 7% to 12% [2].

In advanced-stage NSCLC, doublet combinations of platinum compounds are reference regimens for first-line treatment, with approximately a third of patients obtaining an objective response and another 20% to 30% achieving temporary disease stabilization. After failure of first-line chemotherapy, many patients still have a good performance status and remain candidates to receive further anti-tumour treatment. Two chemotherapeutic agents, docetaxel and pemetrexed, and the biologic drug erlotinib are currently approved for clinical use in the second-line setting, but specifically, a single-agent chemotherapy using docetaxel or pemetrexed is the recommended first option for these patients [5].

Docetaxel is currently one of the most commonly used treatments for patients who have progressed after undergoing previous platinum-based therapy, having shown superior efficacy compared to a number of other regimens and best supportive care (BSC) alone [6,7].

Pemetrexed is a pyrrolopyrimidine-based antifolate cytotoxic drug traditionally used as monotherapy for the second-line treatment of patients with locally advanced or metastatic NSCLC and, in combination with cisplatin, for the treatment of chemonaïve patients with unresectable malignant pleural mesothelioma (MPM). Recently presented data that showed a treatment-by-histology interaction associated with different clinical outcomes has resulted in a change in the NSCLC indication to patients with other than predominantly squamous cell histology. Moreover, the Summary of Product Characteristics (SmPC) of Alimta was extended in April 2008 to include first-line treatment of NSCLC in combination with cisplatin, also in the other than predominantly squamous cell histology group [8].

The clinical benefit of pemetrexed treatment in patients with advanced predominantly non-squamous NSCLC has been consistently demonstrated across three randomized phase III trials. A pre-specified analysis of a first-line study comparing pemetrexed and cisplatin with gemcitabine and cisplatin in NSCLC reported improved overall survival in pemetrexed-treated patients with non-squamous histology compared with those with squamous cell histology [9]. This outcome advantage of pemetrexed in non-squamous histology was confirmed by a retrospective unplanned subgroup analysis of a phase III trial comparing second-line pemetrexed versus docetaxel [10] and by a pre-planned analysis of a phase III trial of platinum-based chemotherapy in the first-line setting, followed or not by pemetrexed maintenance [11]. The efficacy differences that were found, relative to histology type, may give clinicians clues for refining treatment choices and finally achieve the goal of individualised drug therapy for patients with NSCLC.

In the current context of increasingly stretched healthcare budgets, however, efficacy and safety data should not be used as the only criteria to guide therapeutic decisions. The growing needs and demands of the population, leading to increasing use of cancer drugs, and the higher costs of new treatments have forced decision-makers to place greater emphasis on how to use the limited resources in the most efficient way. The economic evaluation of health interventions can assist local or national budget-holders in their resource allocation decisions. It can also provide guidance for medical oncologists by confirming patient subgroups for which pemetrexed may be not only cost-effective, but also have clinically superior outcomes. The aim of our study was to conduct an economic evaluation of pemetrexed versus docetaxel in the second-line treatment of patients with predominantly non-squamous NSCLC based on the findings of the Scagliotti retrospective analysis, as one step forward in the "tailored therapy" approach.

## Methods

### Model Structure

**Figure 1:**
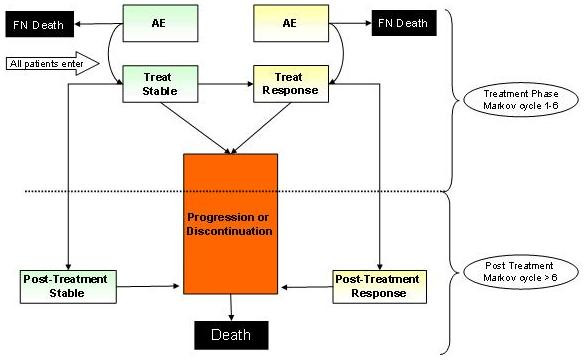
**Model schematic**. Diagrammatic view of the model structure comprising three main health states: Stable, Response and Progression. (FN: febrile neutropenia; AE: adverse event)

The economic model was built in MS Excel^© ^and follows a Markov structure based on three main health states: stable, response and progression. A schematic of the model is shown in Figure 1. The chronology of the model may be split roughly into the treatment phase and the post treatment phase, although patients who enter Progression will discontinue their treatment immediately, while other patients may complete their full course of chemotherapy. This means that some overlap of the two phases exists among the patient cohort. The cycle length is 21 days, and for each model cycle patients face a risk of changing health states to Response, Progression or Death, and also of experiencing a treatment-related adverse event (AE). Patients are permitted up to a maximum of 6 cycles of chemotherapy, after which they will either enter the post treatment states of Stable or Response until they enter Progression. Patients who progress during chemotherapy may also discontinue prior to treatment completion and proceed directly to the Progressive state. Stable and responding patients can either move to Progression or remain in the current state. The model assumes death follows Progression. Patients who have responded and then progress move directly to the Progression state from the Response state. Discontinuation occurs when patients stop chemotherapy treatment. It is assumed that such patients proceed directly to the Progressive state.

In both the Response and Stable states patients have a probability of developing AEs as long as they are in the treatment phase. These AEs include febrile neutropenia (FN), neutropenia, nausea/vomiting, fatigue, diarrhoea, rash and alopecia (hair loss). FN is the only adverse event to carry a risk of death. Discontinuations due to AEs are considered and those who stop active treatment are immediately assumed to progress in their disease.

The study takes the perspective of the Spanish Health Care system focusing on direct medical costs (chemotherapy treatment, AE treatment and BSC). The key comparators for the model are pemetrexed (Alimta^®^) and docetaxel (Taxotere^®^).

### Model Parameters

The patient population under evaluation in this model were stage IIIB or IV patients with NSCLC with predominantly non-squamous histology who had previously undergone a course of chemotherapy and were eligible for second-line therapy. The time horizon was set to three years, which, for this patient cohort, equated to a lifetime model and was confirmed by Spanish clinicians as a suitable timeframe. Discount rates were set to 3% for both costs and benefits to account for the time preference of costs and benefits accrued.

### Model Inputs: Efficacy

All clinical inputs for the predominantly non-squamous population come from the post-hoc retrospective subgroup analysis of the head-to-head JMEI trial [10] [data on file, Eli Lilly JMEI trial 2008], which compared second-line pemetrexed versus docetaxel. This retrospective analysis was the source on which the European Medicines Agency (EMEA) based its decision to change the SmPC indication. Several other randomized trials have reinforced this finding of improved survival in the predominantly non-squamous group including first-line and maintenance therapies [9-11]. The model input variables are efficacy (overall survival, progression-free survival and tumour response), AE rates and AE discontinuations.

Median overall survival was used to determine the risk of death in the progression state. Median overall survival from the trial was chosen as more appropriate than mean values, as mean values would be skewed and rely on assumptions of the survival of those who did not fail during the course of follow-up. The median progression-free survival (PFS) duration was subtracted from the median overall survival duration in order to estimate the median time spent in progression before death. In order to calculate a risk of dying, an exponential distribution was assumed with a constant risk of death, derived from the formula ln(2)/(median time in progression). This risk of death was adjusted to fit a 3-week period, corresponding to the per-cycle risk of death. The exponential distribution is often used for the modelling of failure times and is applicable for use in time-to-event data [12].

PFS was defined as the time from randomization until documented progression or death from any cause and was censored at the date of the last follow-up visit for patients who were still alive and who had not progressed. The PFS data were split out into responders and non-responders to account for the difference in PFS duration achieved by those two groups. Tumour response was assessed using the Southwest Oncology Group criteria and required confirmation at least 4 weeks after initial response. The efficacy inputs are summarised in Table 1. Overall survival for patients with non-squamous NSCLC treated with pemetrexed was statistically superior to that for docetaxel (median 9.3 vs. 8.0 months; HR 0.778, 95% CI 0.607-0.997).

**Table 1:** Clinical efficacy inputs

All patients with non-squamous histology	PEM N = 205	DOC N = 194
Median survival, months^1 ^(95% CI)	9.30 (7.80-9.7)	8.00 (6.30-9.30)

Overall survival hazard ratio (95% CI)^1^	0.78 (0.61-1.00)	

Median PFS, months^1^	3.10	3.00

PFS hazard ratio (95% CI)^1^	0.82 (0.66-1.02)	

Response rates (%) (Complete response + partial response)^2^	10.78	8.81

Response rates (95% CI)	(7.25-15.80)	(5.59-13.66)

Proportion of responders by cycle 2 (%)^3^	45.83	

Source: ^1 ^Scagliotti et al, 2009 [10]; ^2 ^Data on file, Eli Lilly, 2008; ^3 ^Taken from JMEI data, number of responders by cycle 2/total responders (22/48)

PEM: pemetrexed; DOC: docetaxel; CI: confidential interval; PFS: progression-free survival

Table 1 presents the clinical efficacy inputs that drive the model.

### Model Inputs: Adverse Events (AEs)

The most common (> 5%) Grade 3/4 drug-related AEs experienced by patients with NSCLC of non-squamous histology reported in the JMEI study were included in the model. Grades 1/2 drug-related AEs, apart from alopecia, were excluded on the basis that they do not have a major impact on patients' quality of life and costs of treatment. FN was included even if it only occurred in a small number of patients as it is the only AE with a risk of mortality and has significant cost implications. AEs were assumed to be mutually exclusive of one another: very few patients experienced more than one grade 3/4 AE concurrently. The incidences of nausea and vomiting were added to yield a single AE input. It was assumed for all AEs, except alopecia, that they were resolved/treated in the same cycle within which they occurred and the utility decrement was linked to a single cycle duration. For all AEs except FN, a constant risk was assumed.

With respect to FN, it was assumed that most cases would occur after administration of the first cycle of treatment and thereafter, a constant risk (per cycle) was assumed: the FN risk was varied over the Cycles 1, 2 and Cycles 3 and above, and remained constant for each cycle after cycle 3 (Table 2). A mortality risk of 3.9% was set based on mean all-cause mortality data taken from a review of 23 studies on FN covering 4938 patients [13]. One hundred percent of patients are assumed to be hospitalised for grade 3/4 FN.

**Table 2:** Risk of febrile neutropenia across the cycles

Risk of febrile neutropenia	Cycle 1	Cycle 2	Cycle 3+
Risk per cycle with pemetrexed	0.00%	1.55%	1.04%

Risk per cycle with docetaxel	12.11%	2.63%	1.05%

Source: Eli Lilly, data on file, JMEI trial, 2008

The risk per cycle of a patient experiencing FN assuming greatest risk after cycle 1, remaining constant after cycle 3.

The AE rates are shown in Table 3. A significantly higher number of patients in the docetaxel group experienced haematological toxicities such as FN and neutropenia, compared to those in the pemetrexed group. Patients receiving pemetrexed also reported a lower incidence of patient-felt toxicities, like alopecia. These rates are comparable with the AEs reported in the overall NSCLC population in the JMEI study.

**Table 3:** Incidence of Grade 3/4 adverse events

	FN	Neutropenia	Nausea/Vomiting	Fatigue	Diarrhoea	Alopecia
Pemetrexed	2.59%	4.66%	1.55%	5.70%	0.52%	7.25%

Docetaxel	15.79%	41.58%	1.58%	5.79%	1.58%	38.95%

Source: Eli Lilly, data on file, JMEI trial, 2008

FN: febrile neutropenia

Adverse events for the most common Grade 3/4 rates from the predominantly non-squamous population from the JMEI trial, 2008.

Treatment discontinuations due to serious AEs and discontinuations due to patients' wishes were incorporated into the model, these being the two main reasons for stopping treatment. Discontinuation rates for pemetrexed were 7.42% due to serious AEs and 8.68% due to patients' wishes.

### Model Inputs: Costs

Costs included in the model were those related to chemotherapy treatment, AE treatment and BSC (Table 4). The Spanish reference database BOT issued by the General Spanish Council of Pharmacists [14] was used for medication prices, including chemotherapy. Public prices were applied and value-added tax (VAT) included. Hospital treatment costs and laboratory tests were sourced from the most up-to-date references from the healthcare databases Oblikue and SOIKOS [15,16] and expressed in year 2007 values, inflating earlier values where necessary by using published consumer price indices reported by the Spanish National Statistics Institute [17].

**Table 4:** Unit costs

Resource	Price (€)	Source
Dexamethasone 1 mg tablets × 30 (Fortecortin^®^)	2.97	[14]
Folic Acid 400 mcg tablets × 28 (Zolico^®^)	3.39	
Vitamin B12 1 mg vial × 8 (Cromatonbic^®^)	3.12	
Pemetrexed (100 mg vial)	297.35	
Pemetrexed (500 mg vial)	1295.75	
Docetaxel (20 mg vial)	190.09	
Docetaxel (80 mg vial)	604.43	

Complete Blood Count includes white cell differential	16.05	[16]
Biochemical Analysis includes renal function and liver function tests	24.78	

Chemotherapy administration < 2 hours clinic time	135.21	[16]

Neutropenia	2086.13	Expert panel, [18]
Nausea and Vomiting	516.20	
Fatigue	575.30	
Diarrhoea	691.04	
Alopecia (Hair loss)	0.00	
Rash	64.74	
Febrile Neutropenia	3310.85	

BSC costs		Expert panel
Cost of weighted home visit per 21 day cycle	125.83	
Cost of weighted outpatient visit per 21 day cycle	41.64	
Total BSC cost per 21 day cycle	167.47	

Terminal/Palliative care (weighted one-off cost)	23660.91	Expert panel

BSC: best supportive care

Unit costs and sources for all resource used in the model.

The remaining costs for this model were obtained from two sources. The first was an independent opinion-based study by IMS Health Economics and Outcomes Research (HEOR) Spain [Unit Cost and Patterns of Treatment of Adverse Events and Providing Best Supportive care in patients with Stages IIIb and IV Non-Small Cell Lung Cancer. A report from Opinion-Based Survey for Spain. IMS Health, 2008] conducted among an expert panel of 5 oncologists in Spain using a semi-structured questionnaire to describe the local treatment practice and collect data on costs of treatment in NSCLC. The second report is a cost data collection made by IMS Health HEOR Spain to determine AE and BSC costs in patients with advanced NSCLC [Cost of treatment for 1st line non-small cell lung cancer (NSCLC) in Europe. Report on Spain. IMS Health, 2007]

### Model Inputs: Chemotherapy Related Costs

Pre-medication and laboratory tests were based on the SmPC and Spanish clinical practice for both pemetrexed and docetaxel. In line with the SmPC for pemetrexed, patients were required to take oral folic acid, intramuscular vitamin B12 injection and corticosteroid treatment before, during and after treatment with pemetrexed. Docetaxel patients received only corticosteroid treatment. Laboratory tests comprised full blood count with white cell differential, liver function tests and renal function tests applied as per the individual SmPCs.

Chemotherapy unit costs were calculated using a point estimate body surface area of 1.7 m^2^. Resource use for each administration session was a cost of € 135.21 based on less than 2 hours clinic time for administering the chemotherapy [16].

### Model Inputs: Adverse Events Costs

Bottom-up cost data for AEs was not available from published sources. An opinion-based survey conducted among five oncologists in Spain provided information on the treatment algorithms and resource use for each of the grade 3/4 AEs, based on hospitalisation and drug costs. The estimated AE cost is a weighted average of AE costs in four settings: inpatient including hospitalization costs, outpatient, daycare and no treatment; the distribution of patients across these settings was provided by the clinicians.

For fatigue no costs were available, therefore anaemia costs were substituted. No cost was assigned to alopecia. For FN, the results from the Spanish clinician survey were not applied since the field survey reported some impractical assumptions. A flat cost provided by Mayordomo, 2009 [18] was therefore applied instead.

### Model Inputs: Best Supportive Care Costs

BSC costs were split into two types: 1) BSC costs during active treatment, post treatment and progression; and 2) terminal/palliative care costs. In the model, BSC costs were applied during all three phases (active treatment, post treatment and at progression) but the cost per cycle for BSC was reduced by 50% during the active treatment phase to reflect the likelihood that less intensive care is required during this period. Applying less intensive BSC during active treatment is a recognised practice in Spain and is supported in the Spanish literature [19].

BSC during active treatment, post treatment and progression was calculated based on outpatient visits and home visits. The distribution and frequency of visits to the oncologist, GP, nurse and physiotherapist were obtained from the opinion-based survey conducted among oncologists in Spain. Resource-use identified in this survey was multiplied by the unit costs for services by these healthcare professionals to estimate a cost of BSC. Unit costs for GP visits, nurse visits, physiotherapist visits and oncologist visits were obtained from the Oblikue database [16]. These costs per setting values were then weighted according to the proportion of patients receiving BSC in each setting. The weighted cost for outpatient and home visits was calculated at € 241.90 per month, or € 167.47 per 21-day cycle. For the active treatment phase, this cost was reduced by 50% to € 83.74 and the reasons for this are discussed below.

The terminal/palliative care cost consisted of a separate one-off cost based on the opinion-based survey among oncologists. Costs for hospital (inpatient), hospice (inpatient) and nursing home were multiplied by the average estimated number of days each patient would spend in each setting to obtain a total palliative care cost. The cost per day in hospice and the cost per day in nursing home, which were not available from Spain, were calculated based on the ratio of these costs in the UK relative to a day in hospital in the UK. The same ratio was then applied to the cost of a day in hospital in Spain.

A total cost of terminal/palliative care cost was calculated at € 23,660.91. All patients that entered the terminal/palliative care phase were assumed to be extensively hospitalised, that is, they would each spend some time in hospital, in hospice or in a nursing home. The impact of patients spending different proportions of time in these treatment settings, such as at home, is explored in the sensitivity analysis.

The model uses a chemotherapy cost of € 2203 for pemetrexed and € 963 for docetaxel per patient per cycle based on an assumption of no wastage of the unused portion in the vials, that is, utilising per mg costing. In the sensitivity analysis, the impact of wastage through discarding the remainder of chemotherapy agents left in the vial was investigated. The total cost per cycle inclusive of premedication, chemotherapy, laboratory and administration costs was € 2384 for pemetrexed and € 1144 for docetaxel (Table 5).

**Table 5:** Summary cost of chemotherapy, administration, premedication and laboratory test costs per treatment option per 21 day cycle

	Pemetrexed 500 mg/m^2 ^(€)	Docetaxel 75 mg/m^2 ^(€)
Chemotherapy costs (based on no wastage)	2202.78	963.31
Administration costs	135.21	135.21
Dexamethasone 1 mg tablets × 30 (Fortecortin^®^)	2.38	4.75
Folic Acid 400 mcg tablets × 28 (Zolico^®^)	2.54	-
Vitamin B12 1 mg vial × 8 (Cromatonbic^®^)	0.13	-
Complete Blood Count includes white cell differential	16.05	16.05
Biochemical Analysis includes renal function and liver function tests	24.78	24.78

**Total cost per cycle**	**2383.87**	**1144.10**

Costs per 21 day cycle for pemetrexed and docetaxel comprising chemotherapy, administration, premedication and laboratory test costs.

### Model Inputs: Utility Values

Utility weights assigned to each health state were based primarily on a societal valuation study of 100 participants rating health states using the standard gamble technique [20]. A mixed model analysis was conducted based on the data collected and values obtained for the health states Stable, Response and Progression. A decrement in utility was also obtained for each of the AEs, which are then applied to either the Stable or Response state, depending on the health state which the patients currently occupy. Table 6 shows the mean utility values derived for specific health states.

**Table 6:** Utility values for the health states with/without adverse events

Disease stage	Adverse Events within each category	Mean utility values
Stable disease	No AE	0.65
	Grade 3/4 Rash	0.62
	Grade 3/4 Alopecia	0.61
	Grade 3/4 Fatigue	0.58
	Grade 3/4 Nausea & Vomiting	0.61
	Grade 3/4 Diarrhoea	0.61
	Grade 3/4 Febrile Neutropenia	0.56
	Grade 3/4 Neutropenia	0.56

Responding disease	No AE	0.67
	Grade 3/4 Rash	0.64
	Grade 3/4 Alopecia	0.63
	Grade 3/4 Fatigue	0.6
	Grade 3/4 Nausea & Vomiting	0.62
	Grade 3/4 Diarrhoea	0.63
	Grade 3/4 Febrile Neutropenia	0.58
	Grade 3/4 Neutropenia	0.58

Progressive Disease	-	0.47

AE: adverse events; BSC: best supportive care

Mean utility values for each health state, incorporating AE utility decrement.

### Sensitivity Analyses: Univariate

A series of univariate sensitivity analyses were conducted to determine the key drivers of cost-effectiveness in the model. These one-way sensitivity analyses consider the variation in the incremental cost, incremental benefit and ICER outcomes when viable ranges of parameter values were independently modified. The following parameters were varied either according to a fixed range based on available data (such as 95% Confidence Interval (CI)) or according to a proportional change from the base case value (Table 7).

**Table 7:** Univariate sensitivity analysis parameters and ranges

Univariate Sensitivity Parameter	Min	Max
Discount rate (baseline = 3% for costs, 3% for benefits)	0%	6%

All costs (excluding chemotherapy drugs) varied by ± 25% (baseline = 100%)	75%	125%

Chemotherapy drugs varied by ± 25% (baseline = 100%)	75%	125%

All costs varied by +/- 25% (baseline = 100%)	75%	125%

Per vial costing with 100 mg and 500 mg pemetrexed vials available	-	-

Mean body surface area (BSA) in m^2^	1.6	2

Chemotherapy administration time varied +/- 50% (baseline = 100%)	50%	150%

Hospital days for AE varied +/- 50% (baseline = 100%)	50%	150%

Exclude BSC costs for treated patients once they complete treatment or enter progression	-	-

Cost of febrile neutropenia varied by +/- 25% (baseline = 100%)	€ 2483	€4139

Disutility assigned to AEs varied +/- 50% (baseline = 100%)	50%	150%

Assume no disutility assigned to AEs (so only have a cost impact in model)	-	-

Utility weights assigned to health states varied between upper and lower 95% CI	0.60 ^stable/response^ 0.50^with an AE^	0.72^stable/response^ 0.66^with an AE^

95% CI for response rate for PEM (base case = 10.78%)	7.25%	15.80%

95% CI for response rate for DOC (base case = 8.81%)	5.59%	13.66%

95% CI for survival hazard ratio for PEM vs. DOC (base case = 0.778)	0.607	0.997

95% CI for progression free survival hazard ratio for PEM vs. DOC (base case = 0.823)	0.664	1.020

Assume weighted terminal/palliative care cost	€6927	-

Model time horizon set to 1 year (baseline = 3 years)	-	-

Setting cost of fatigue to zero	-	-

Duration of treatment (no. of cycles; base case = 6)	4	5

AE: adverse event; BSC: best supportive care; CI: confidential interval; PEM: pemetrexed; DOC: docetaxel

A univariate sensitivity analysis was conducted to test which parameters the cost-effectiveness ratio was sensitive to. Table 7 shows the ranges within which each parameter was varied.

### Sensitivity Analysis: Probabilistic

A probabilistic sensitivity analysis was conducted in the model to take account of the simultaneous effect of second-order uncertainty relating to parameter values. CIs around the median statistics for these parameters were determined based on an assumed distribution of the exponential function for time-to-event data, and beta distribution for risks or rates. Utility values were also assumed to have a beta distribution, with their standard errors obtained from 2000 iterations of the mixed model used to obtain the utility values. These were used to draw repeated samples for the median overall survival, PFS for non-responders, PFS for responders, utility values and treatment discontinuation rates. Each simulated group of samples produced a result. These simulations were repeated over 1000 iterations to create a cost-effectiveness plot of likely outcomes, and a cost-effectiveness acceptability curve (CEAC) to illustrate the probability of achieving cost-effectiveness given a range of willingness-to-pay thresholds.

## Results

### Cost-effectiveness Results

The base case cost-effectiveness analysis was run over a 3-year time-horizon, assuming no wastage, with BSC provided during treatment, post treatment and at progression. Patients received a mean of 3.81 pemetrexed treatment cycles and 3.82 docetaxel treatment cycles out of a possible maximum of 6 cycles. The comparison of pemetrexed versus docetaxel resulted in an ICER of € 23967 per QALY and € 17225 per LYG. In this comparison, pemetrexed was found to have a total benefit of 0.52 QALYs compared to 0.42 QALYs for docetaxel. In terms of LYG, pemetrexed reported 1.03 LYG compared to 0.89 for docetaxel, an incremental gain of 0.14 life years. A summary of the results are contained in Tables 8 and 9.

**Table 8:** Summary of costs and benefits

	Pemetrexed	Docetaxel	Incremental difference
Total Cost (€)	€ 34677	€ 32343	€ 2334

**Cost Breakdown**			
Chemotherapy cost	€ 8721	€ 3997	€ 4724
Treatment (admin/premeds)	€ 691	€ 690	€ 1
AE cost	€ 371	€ 2891	-€ 2520
BSC cost	€ 2404	€ 1900	€ 503
Terminal cost	€ 22491	€ 22865	-€ 374

**Total Benefit**			
QALYs	0.52	0.42	0.10
LYG	1.03	0.89	0.14

AE: adverse event; BSC: best supportive care; QALY: quality-adjusted life year; LYG: life year gained

Table 8 summarises costs and benefits in terms of QALYs and LYG for both pemetrexed and docetaxel. The incremental difference between both treatment arms is also presented.

**Table 9:** Incremental cost-effectiveness ratio results

ICER	Pemetrexed vs Docetaxel
ICER (QALYs)	€ 23967

ICER (LYG)	€ 17225

ICER: incremental cost-effectiveness ratio; QALY: quality-adjusted life year; LYG: life year gained

The ICER for pemetrexed versus docetaxel demonstrates that pemetrexed is a cost-effective treatment compared to docetaxel.

The results indicate that whilst chemotherapy costs are higher for pemetrexed than docetaxel, the costs of adverse events are less. Lower AE costs are a result of less toxicity for pemetrexed compared to docetaxel. In particular, neutropenia and febrile neutropenia which incur significant costs in Spain occurred with a notable difference between pemetrexed and docetaxel. The pemetrexed arm reported a 4.66% rate of neutropenia, while docetaxel reported a 41.58% rate; for febrile neutropenia the rates were 2.59% and 15.79%, respectively.

### Modelled Survival Curves

**Figure 2:**
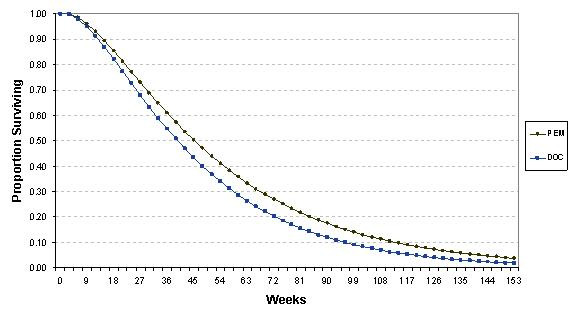
**Overall survival curve; model output**. Modelled survival curve using data from the JMEI trial demonstrates that pemetrexed has improved overall survival compared to docetaxel. (PEM: pemetrexed; DOC: docetaxel)

Data from the key trial was fitted to the model with an exponential distribution in order to parameterise the survival function which assumed a constant hazard rate. The survival curves demonstrated that pemetrexed showed superior overall survival compared to docetaxel (Figure 2).

### Univariate Sensitivity Analyses

**Figure 3:**
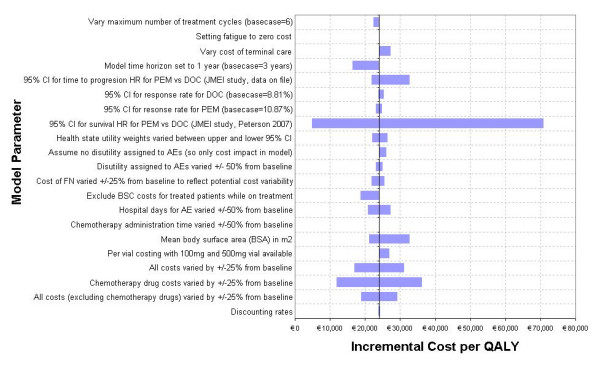
**One way sensitivity analysis tornado diagram ICER results**. CI: confidence interval; PEM: pemetrexed; DOC: docetaxel; AE: adverse events; FN: febrile neutropenia; BSC: best supportive care Results from the one way sensitivity analysis demonstrate that the model is primarily sensitive to the 95% CI for the survival hazard ratio comparing pemetrexed versus docetaxel.

The tornado diagram (Figure 3) shows that the model is sensitive to one key clinical parameter, overall survival, which drives the cost-effectiveness. Running the model using the 95% CI intervals for the survival hazard ratio for pemetrexed versus docetaxel had the effect of changing the incremental cost-effectiveness ratio (ICER) considerably - at the lower limit of the hazard ratio, indicating a longer overall survival with pemetrexed, the ICER decreased to € 4891 whilst at the upper limit of the hazard ratio indicating a shorter overall survival for pemetrexed, the ICER increased to € 70768, showing a higher cost but lower benefit.

The other key drivers of the model are the cost of chemotherapy drugs, BSC costs during the active treatment phase and the time horizon of the model. Reducing the cost of the chemotherapy drugs improves the cost-effectiveness of pemetrexed compared to docetaxel, whilst excluding BSC costs for patients on treatment reduced the ICER to € 18799. Setting the time period of analysis to one year instead of three reduces the ICER to € 16373. This is due to the treatment costs of AEs being accrued in the first year alongside chemotherapy. A major cost-offset is realised by the AE treatment costs, which are eroded with the consideration of best supportive/palliative care costs in the ensuing years.

In the model, patients are assumed to spend most of the time admitted to a hospital or institution during terminal care. This assumption was tested by using statistics provided by Gómez-Batiste et al for the proportion of patients dying in different settings, which state that 42% of patients died at home, 41% in a conventional hospital ward, and 17% in a palliative care centre [21]. This brought the one-off cost of terminal care down to € 4600, but the ICER remained similar to the baseline at approximately € 27000.

### Probabilistic Sensitivity Analyses

**Figure 4:**
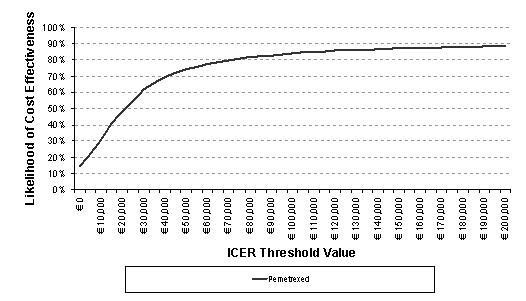
**CEAC, cost per QALY pemetrexed compared to docetaxel as the reference**. CEAC: cost-effectiveness acceptability curve; QALY: quality-adjusted life year; ICER: incremental cost-effectiveness ratio. The cost effectiveness acceptability curve demonstrates the likelihood of pemetrexed being cost-effective compared to docetaxel at each ICER threshold value for cost per QALY and cost per LYG.

**Figure 5:**
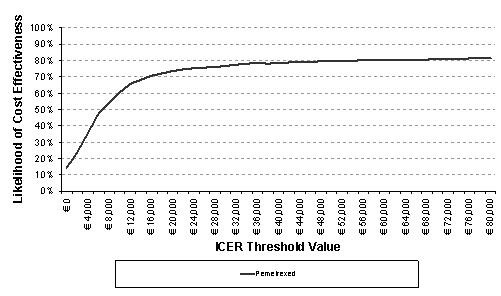
**CEAC, cost per LYG pemetrexed compared to docetaxel as the reference**. CEAC: cost-effectiveness acceptability curve; QALY: quality-adjusted life year; ICER: incremental cost-effectiveness ratio

The results of the PSA correlated the base case results for each of the patient population groups. The cost-effectiveness acceptability curves (CEACs) below show the likelihood of pemetrexed being cost-effective compared to docetaxel when considered across a range of thresholds for the cost per QALY and per LYG (Figure 4 and Figure 5).

The CEAC plot shows that pemetrexed has a 62% likelihood of having a cost per QALY below € 30000 and a 77% likelihood of having a cost per LYG below € 30000. These thresholds have been selected based on Sacristan et al and Ortún et al [22,23].

**Figure 6:**
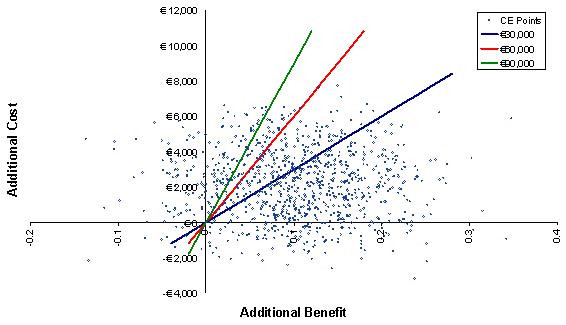
**cost-effectiveness plot, cost per QALY**. QALY: quality adjusted life year; CE: cost-effectiveness The majority of simulations from the PSA are in the top right quadrant demonstrating the confidence limits that surround the base case scenario. Threshold values at € 30000, € 60000 and € 90000 have been added to show the number of iterations that fall within each ICER threshold.

The cost-effectiveness plot (Figure 6) indicates the confidence limits that can be placed around the base case. The plot data from the PSA demonstrates that the majority of simulations (78%) resulted in additional costs and benefits for pemetrexed over docetaxel (the top right quadrant). The probability that pemetrexed would dominate docetaxel - less costs and additional benefit - is 13%. There is only a small likelihood (1%) that pemetrexed would have lower cost and less benefits than docetaxel, whilst there is an 8% probability that pemetrexed would have additional cost and less benefit (top left hand quadrant of the plot). This figure also illustrates that the majority of simulations fall under the € 30000 per QALY threshold - 62% of the simulations fall below the € 30000 per QALY threshold, whilst 77% fall under € 60000 per QALY and 83% under a € 90000 per QALY threshold.

## Discussion

New regimens for the treatment of NSCLC aim to increase the objective tumour response and survival rates as well as to reduce toxicity, decrease symptoms and improve psychological well being for patients. In inoperable advanced second-line NSCLC, active treatment is well established in Spain and achieves both palliation of symptoms and improvement of QoL in addition to prolonging survival.

The results of the model show that pemetrexed produces better outcomes and at an increased cost. At € 23967, the cost per additional QALY for pemetrexed compared to docetaxel is well within the € 30000 threshold for QALY discussed by Sacristan and Ortún [22,23]. The ICERs show that the additional cost incurred by pemetrexed is justified by the gain in outcomes.

Pemetrexed is associated with a further increase in overall survival of 0.14 years compared to the current standard of docetaxel in the predominantly non-squamous histology subgroup, and moreover shows considerable decrease in reported AEs. The avoidance of treatment-related AEs contributes significantly to the cost-offset accomplished by using pemetrexed and contributes favourably to the health-related QoL of the patients undergoing active chemotherapy.

Three randomised phase III trials have consistently demonstrated the clinical benefit of pemetrexed treatment in patients with predominantly advanced non-squamous NSCLC [9-11]. These findings resulted in a specific change to the SmPC indication for pemetrexed in April 2008 to the second-line treatment of patients with other than predominantly squamous cell histology as well as introducing an additional indication for first-line therapy in combination with cisplatin in this predominantly non-squamous patient group.

Another key consideration contributing to the cost-effectiveness of pemetrexed is the difference in toxicity profile, especially in severe and expensive AEs such as neutropenia and FN. The differences in the event rates for these two AEs are remarkable. For FN pemetrexed reports a 3% rate as opposed to 16% for docetaxel, and for neutropenia the difference is even more pronounced at 5% and 42%, respectively. The impact of these events is evidenced by the lower ICER when the time horizon is shortened to a period of one year. At one year, the key cost differences come from chemotherapy drug prices and AE costs because best supportive care and terminal care costs have yet to be accrued in sufficient amounts. Pemetrexed remaining cost-effective with such a short time horizon indicates that the cost-difference in drug prices has been more than made up for with the difference in AE-related costs.

AE costs were not obtained from published sources in the literature since such costs in the specific context of second-line NSCLC patients were not available for the breadth of AEs included in this model. FN and neutropenia costs were particularly important given their significant difference in rates between pemetrexed and docetaxel. The cost of FN was the only one available from literature [18], based on a retrospective chart review and adjusted specifically for the context of lung cancer. About half of the neutropenia patients are treated as outpatients or day cases, and the medication used in treating neutropenia must be costed separately. These include granulocyte colony growth factors (G-CSF), which have significant cost. The exact proportion of patients treated with G-CSF in the trial for the non-squamous population is unavailable; however, the opinion-based survey from which the resource use of AEs were derived indicates that a majority of clinicians in Spain would use G-CSF in treating Grade 3/4 neutropenia. The weighted average cost of neutropenia is approximately two-thirds the cost of an episode of FN, making it a realistic estimate, given the likelihood that neutropenia involves less intensive forms of treatment than FN, distributed among different settings..

BSC was applied at all three stages - during treatment, post treatment and at progression - though the cost of BSC during the active treatment phase was reduced by 50% to reflect a reduced need for BSC while the patient was receiving chemotherapy. This reduction in BSC cost was included to reflect the assumption that less intensive BSC is required while patients are on active therapy [19]. Whilst including BSC at all three levels increases the overall ICER, it is reflective of current practice in Spain, since most patients receive continuous care throughout their illness. On average, pemetrexed had a higher BSC cost than docetaxel. This is attributable to the greater overall survival of pemetrexed patients, who spend more time in the Progression state accruing post-treatment BSC costs. Eliminating BSC during the treatment phase was examined as a scenario in the sensitivity analysis and it had the effect of reducing the ICER to € 18799 per QALY. Both AE and BSC costs were estimated from opinion-based surveys of five Spanish clinicians and hence may be considered a limitation of the study.

The base case setting reflected current practice in Spain. It included costs and benefits set at a discount of 3% each and the assumption that left over cytotoxic agents in vials were reused to minimise wastage as would be normal practice in most large oncology centres. A scenario was also run to reflect the possibility of wastage, as may occur in smaller centres. This had a small effect of increasing the ICER to € 26741, still below the accepted threshold.

The univariate sensitivity analysis demonstrated that the clinical parameter, overall survival, is by far the key driver of the model. When improved survival rates for pemetrexed are applied using the lower 95% CIs of the survival hazard ratio, the ICER is significantly reduced to € 4891 whilst, conversely, using the upper limits of the hazard ratio increased the ICER to € 70768. Similarly, adjusting the PFS for pemetrexed using the 95% CIs of the hazard ratio also had a similar, but less pronounced effect - the impact is greatest at the higher end of the CI with a shorter PFS for pemetrexed, raising the ICER to € 32549, just over the € 30000 limit. Both these clinical parameters have an impact on the rate of death, which is where the greatest benefit may be identified. Overall survival directly impacts the calculation of the risk of death from progression, and the length of PFS acts to delay patients from entering the Progression state, which is the only state from which patients may die, other than febrile neutropenia. To this end, the rate of neutropenia-related death was also varied to 0% in the model, but this was found to have a minimal impact.

The model was also sensitive to the cost of chemotherapy drugs: an increase of 25% in the cost of cytotoxics increased the ICER to € 36096. An increase in the drug acquisition costs acts to increase the overall ICER: proportional increase implies a larger cost difference of pemetrexed relative to docetaxel to be compensated for by savings in such items as reduced toxicity.

The results of the PSA further substantiate the conclusion that pemetrexed is a cost-effective alternative to docetaxel. Of the 1000 simulations run, 92% reported that pemetrexed will have additional benefit compared to docetaxel - 78% of the simulations indicated higher cost and additional benefit as demonstrated in the north-east quadrant of the cost-effectiveness plot, whilst a further 13% suggested that pemetrexed would dominate docetaxel with lower costs and additional benefits. Together, these far outweigh the 8% probability that pemetrexed has additional costs and fewer benefits than docetaxel and provide further evidence to endorse the use of pemetrexed in this non-squamous histology group of patients with NSCLC.

The cost-effectiveness acceptability curve demonstrates that the likelihood of the cost per QALY falling below € 30000 is 62% and of the cost per LYG is 77%. These values can provide a degree of confidence in the probability of pemetrexed being a cost-effective treatment option.

In Spain docetaxel is considered to be the most likely alternative to pemetrexed and therefore is the main comparator. Currently, no head-to-head trial data exist to compare pemetrexed to an intervention other than docetaxel. An indirect comparison would therefore be required in order to derive clinical data inputs for other comparators. This indirect comparison is currently not feasible against erlotinib and BSC, as no clinical evidence using these two interventions is reported specifically for the predominantly non-squamous population. The model does, however, have the capacity to add relevant clinical inputs for these comparators at a future point in time if either direct comparison data or data to allow an indirect comparison become available.

A recent cost-utility analysis has however been published comparing erlotinib, docetaxel, pemetrexed and BSC in patients with advanced NSCLC without distinguishing histology sub-type who had failed previous chemotherapy regimens [24]. No difference in efficacy between pemetrexed and erlotinib was noted - both reported a QALY gain of 0.24 years and a LYG of 0.77, but with cost-savings of € 9479 in favour of erlotinib. This analysis was based on an indirect comparison of the treatments over a 2 year time horizon using survival results from three studies: Shepherd 2000, Hanna 2004 and Shepherd 2005 [7,25,26]. This analysis did not differentiate between histology subgroups within the NSCLC group as data were only available for the overall NSCLC population, and it therefore does not account for pemetrexed's efficacy benefit seen within the predominantly non-squamous histology subgroup analysed in this paper.

While no clinical data are reported for a head-to-head comparison of pemetrexed with BSC, there are other economic evaluations that have established the cost-effectiveness of second-line chemotherapy compared to BSC in the second-line treatment of NSCLC [27,28]. These studies have found that monotherapy docetaxel is a cost-effective alternative to BSC. These other studies allow us to place the current study in the context of other therapies that have been evaluated for their cost-effectiveness.

The model did not take into account dose reductions or delays in treatment since these estimates are already accounted for in the phase III trial survival estimates. The model also assumes the duration of therapy is linked to AE discontinuation rates and tumour progression. These data were available from the phase III RCT. The maximum number of treatment cycles in the base case was set to 6, which was endorsed by Spanish clinical experts; however, the trial results indicate that the median number of cycles administered was approximately four cycles for both treatment arms.

This economic model contains several limitations. Given the choice of perspective, that of the Spanish healthcare system, indirect and non-medical costs were not included in this model. Consideration of a societal perspective incorporating productivity loss may increase the burden associated with the disease and the associated side-effects of the treatment, thus making the alleviation of symptoms and reduced toxicity even more cost-effective. It is unclear how using other methods of incorporating the societal perspective will impact the cost-effectiveness of pemetrexed in second-line NSCLC.

Another limitation of this model was the absence of data to show what effect the third-line therapy choices may have had on the reported efficacy results. Hanna et al [25] states that 47% of patients receiving pemetrexed and 37% of patients receiving docetaxel were ultimately treated with third-line therapies; the numbers reported for the non-squamous group are very similar at 48.3% for those receiving pemetrexed and 37.1% for those receiving docetaxel [data on file, Eli Lilly JMEI trial 2008]. This indicates that there may have been a possible confounding factor in the survival advantage attributed to pemetrexed. However, it should also be noted that the safety profile of pemetrexed resulted in a greater proportion of patients whose health status was good enough at the conclusion of second line therapy to receive further anti-cancer care.

The choice of an exponential distribution may also be a limitation of this model. The exponential distribution assumes a constant hazard rate, which is rarely the case in real-life survival. There are alternative distributions that could be used, but it is unlikely that any parameterised distribution will perfectly fit the survival curve output of the trial. Alternative distributions, such as the Weibull distribution, require additional steps in the application to an economic model without adding much value to accuracy in the ultimate result.

## Conclusions

In the Spanish setting, pemetrexed for the second-line treatment of patients with NSCLC other than predominantly squamous cell histology is indicated as a cost-effective chemotherapy option compared to the standard docetaxel, based on its superior overall survival benefit and toxicity profile.

## Competing interests

YA and EW are employees of IMS Health who have received consultancy fees from Eli Lilly and company; AV, KT, JA, JAS, and TD are employees of Eli Lilly and company. CC does not have any conflict of interest to declare.

## Authors' contributions

YA and EW developed the economic model, reviewed and collected data, performed the analyses and drafted the manuscript. AV and KT contributed to the development of the economic model, reviewed and collected data, interpreted the results and drafted the manuscript. CC provided clinical input, validated the model assumptions and helped to draft the manuscript. JA and TD contributed to the conception and design of the original model and revised the manuscript. AC and JAS aided in defining the clinical and economic context and revised the manuscript. All authors read and approved the final manuscript.

## Pre-publication history

The pre-publication history for this paper can be accessed here:

http://www.biomedcentral.com/1471-2407/10/26/prepub
